# 973. Utilization of Project ECHO for COVID-19 Medical Knowledge and Best Practices for Health Professionals serving an Underserved Population

**DOI:** 10.1093/ofid/ofab466.1168

**Published:** 2021-12-04

**Authors:** Natalia Rodriguez, Melanie Goebel, Sheena Bhushan, Shital Patel

**Affiliations:** Baylor College of Medicine, Houston, Texas

## Abstract

**Background:**

During the global COVID-19 pandemic, the release of research and data particularly to guide clinical care evolved rapidly and highlights the critical need for timely, and equitable access to medical knowledge and best practices. Specialized medical knowledge has historically been confined to specialists in academic medical centers and disconnected from healthcare professionals in underserved areas. It is important to bridge this gap and democratize knowledge through a model that supports rapid dissemination of best practices to build capacity in areas of need.

**Methods:**

A Project ECHO partnership was implemented between academic infectious diseases specialists and local healthcare professionals involved in COVID-19 screening, diagnosis and management serving an underserved population. BCM COVID-19 ECHO supported the Access2Health SmartPod COVID-19 clinical operations staffed by a charitable community organization. The SmartPod clinical team were engaged in weekly one-hour ECHO sessions with didactic presentations and case discussions on diverse COVID-19 topics. The program was evaluated at 6 months.

COVID 19 ECHO Model

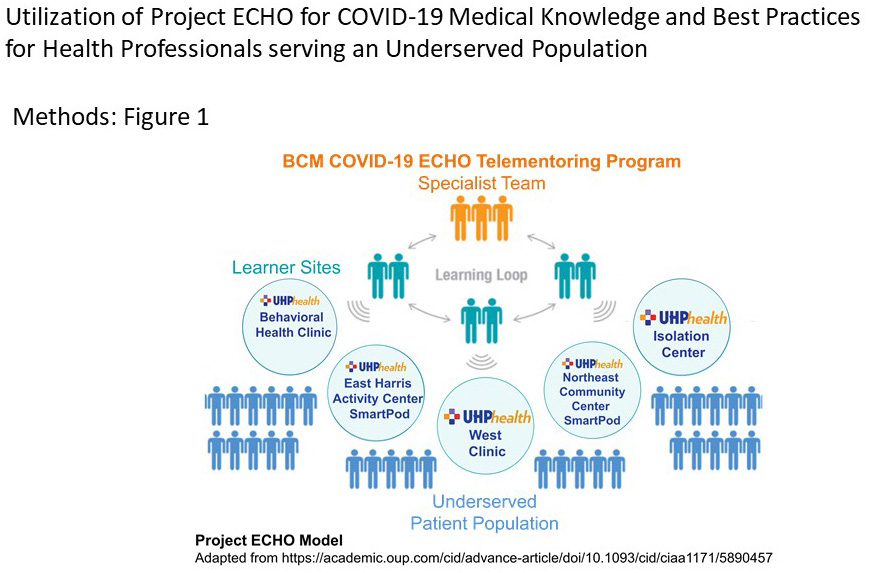

BCM COVID-19 ECHO Telementoring Program with the United Health Partners in the community

BCM COVID-19 ECHO Telementoring Session Topics

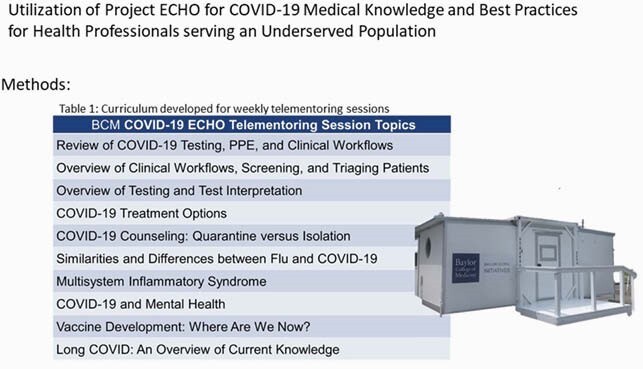

Curriculum developed for the health professionals seeing patients in the SmartPOD and clinics in underserved communities.

**Results:**

In Fall 2020, BCM COVID-19 ECHO facilitated 10 sessions with an average attendance of 8 healthcare professionals per session. Evaluation results indicated high levels of satisfaction with session content and telementoring partnerships, with 80% expressing intent to apply the knowledge and skills acquired from the sessions to their clinical practice.

**Conclusion:**

The Project ECHO model successfully engaged healthcare professionals in a continuous learning loop. With the rapid and vast amount of information during the COVID-19 pandemic, it is important to ensure health professionals have equitable access to medical knowledge and feel empowered to implement best practice changes.

**Disclosures:**

**All Authors**: No reported disclosures

